# Possibilities for Methanogenic and Acetogenic Life in Molecular Clouds

**DOI:** 10.3390/life14111364

**Published:** 2024-10-24

**Authors:** Lei Feng

**Affiliations:** 1 Key Laboratory of Dark Matter and Space Astronomy, Purple Mountain Observatory, Chinese Academy of Sciences, Nanjing 210023, China; fenglei@pmo.ac.cn; 2 School of Astronomy and Space Science, University of Science and Technology of China, Hefei 230026, China; 3 Joint Center for Particle, Nuclear Physics and Cosmology, Nanjing University—Purple Mountain Observatory, Nanjing 210093, China

**Keywords:** origin of life, LUCA, molecular cloud

## Abstract

According to panspermia, life on Earth may have originated from life forms transported through space from elsewhere. These life forms could have passed through molecular clouds, where the process of methanogenesis could have provided enough energy to sustain living organisms. In this study, we calculate the Gibbs free energy released from synthesizing hydrocarbons for methanogenic (acetogenic) life in a molecular cloud, with methane (acetic acid) as the final metabolic product. Our calculations demonstrate that the chemical reactions during methanogenesis can release enough free energy to support living organisms. The methanogenic life may have served as the predecessor of life on Earth, and there is some preliminary evidence from various molecular biology studies to support this idea. Furthermore, we propose a potential distinguishing signal to test our model.

## 1. Introduction

In a recent paper, the authors found that the last universal common ancestor (LUCA) appeared 4.2 billion years ago, just 400 million years after the formation of Earth [1]. This may be a hint of panspermia. According to panspermia [2] and local panspermia [3], life on Earth could have descended from forms of life transported through space. Such life forms could have passed through and survived within molecular clouds. Hydrogen molecules maintain a liquid state within the temperature range of 13.99 K to 20.27 K, which coincidentally coincides with the typical temperature of molecular clouds. Although it is possible to assume the liquid hydrogen environment of molecular cloud life, more severe problems arise correspondingly. So, we do not discuss such a possibility here. Such an extremely low-temperature environment may have unexpected benefits. In Ref. [4], the author argues that the ultra-low temperature environment of molecular clouds may be the reason for the chiral polymer chain of biological molecules. For more information about the effects of low temperatures on the origin of life, see Refs. [5,6]. Additionally, the interstellar medium may harbor pre-biological compounds that foster life-sustaining processes. Although organic molecules like amino acids remain undiscovered in molecular clouds, the presence of numerous amino acids in meteorites [7,8,9,10], which form in molecular clouds, suggests the plausible existence of amino acids within molecular clouds.

A crucial question arises concerning the energy acquisition strategies employed by the cloud lifeforms. The author of this article previously suggested a bioenergetic mechanism driven by cosmic rays [11], which relies on the ionization of hydrogen molecules as its primary energy source. However, alternative energy acquisition pathways that may support life in these extreme environments are worth exploring.

Metabolic activity and reproduction of biological systems require energy transformations through respiration processes, such as aerobic and anaerobic respiration. Aerobic and anaerobic respiration are all redox reactions of biological fuels in the presence of an inorganic electron acceptor. The difference between them is whether the electron acceptor is oxygen. Such redox reactions release Gibbs free energy and produce large amounts of energy stored in ATP.

On Earth, methanogenic bacteria reduce ${\mathrm{CO}}_{2}$ to ${\mathrm{CH}}_{4}$ and release Gibbs free energy needed for life through methanogenesis, which is anaerobic respiration with methane as the final product of metabolism and only found in the domain Archaea. C.P. McKay and H.D. Smith explored the biochemical reactions of hypothetical methane-based life on Titan, with reactants including $C_{2}H_{2}$, $C_{2}H_{6}$, and organic haze [12]. Given the presence of similar compounds in molecular clouds, it is plausible to consider methanogens thriving in these cosmic regions as well. Here, we discuss such probability and calculate the release of free energy for methanogenic life in the environment of molecular clouds. Since acetogenic life has the same electron donor and acceptor through the Wood–Ljungdahl pathway on Earth, the energy release of such biochemical processes in molecular clouds is also discussed in this article. Then, we discuss the relationship between molecular cloud life and LUCA.

This paper is organized as follows: In Section 2, we explore the possible biochemical reactions of methanogenic and acetogenic life in molecular clouds and calculate the released Gibbs free energy. A possible distinguishing signal is also presented in this section. The relation between such methanogenic and acetogenic life in molecular clouds and LUCA is discussed in Section 3. The conclusions are summarized in the final section.

## 2. Possibilities for Methanogenic Life in Molecular Clouds

Methanogens on Earth maintain their activity through methanogenesis, a form of anaerobic respiration. Carbon dioxide is the terminal electron acceptor in methanogenesis through the following chemical reaction:

(1) $${\mathrm{CO}}_{2}+4H_{2}\to {\mathrm{CH}}_{4}+2H_{2}O.$$

With the same electron acceptor and donor, acetogenic processes are also possible through the following reaction:

(2) $$2{\mathrm{CO}}_{2}+4H_{2}\to {\mathrm{CH}}_{3}\mathrm{COOH}+2H_{2}O.$$

As the ${\mathrm{CO}}_{2}$ in molecular clouds is more abundant in the solid phase than in the gas phase [13,14,15], solid ${\mathrm{CO}}_{2}$ provides abundant carbon sources and energy for metabolic activities. Then, life forms may congregate around solid carbon dioxide. The triple point of carbon dioxide occurs at a temperature of 216.58 K and a pressure of 5.11 atm. At temperatures below this threshold, solid carbon dioxide can sublimate directly into a gaseous state as the temperature rises. This transition temperature notably decreases with a reduction in pressure. The temperature is sufficiently low in the place where solid carbon dioxide has been detected. Conversely, in regions where stars are forming, the temperatures are considerably higher, making it challenging to sustain solid carbon dioxide.

The most abundant molecule in molecular clouds is H2, accounting for approximately 70% of the composition. It is also the main collision target of other molecules therein. The second most abundant molecule in the molecular cloud is CO and its hydrogenation reaction, as shown in Equation (3), which could be the energy source of methanogen-like creatures.

(3) $$\mathrm{CO}+3H_{2}\to {\mathrm{CH}}_{4}+H_{2}O.$$

In addition, we also consider the chemical reaction given in Ref. [12], which is

(4) $$C_{2}H_{2}+3H_{2}\to 2{\mathrm{CH}}_{4}.$$

### 2.1. The Calculation of Gibbs Free Energy

The Gibbs free energy released from chemical reactions at temperature *T* and 1 atm can be calculated using the free energies of formation [16,17], which is

(5) $$\Delta G^{\circ }=\Delta H-T\Delta S,$$

where ΔH (ΔS) is the difference in heats of formation (entropy) of product and reactant under standard conditions, which are shown in Table 1.

The free energy between any pressures and 1 atm is given by

(6) $$\Delta G=\Delta G^{\circ }+RT\ln\left(Q\right),$$

where *R* is the universal gas constant and *Q* is the ratio of the activities between products and reactants raised to the power of its multiplying constant in the chemical reaction equation. Following Refs. [12,18], the chemical activities of the gas molecules we use in this article are approximately equal to their partial pressure, which is proportional to the gas mole fraction in the mixture. The activity of solid carbon dioxide is unity. For example, for the C2H2 reaction,

(7) $$Q={\left[pCH_{4}\right]}^{2}/\left[pC_{2}H_{2}\right]{\left[pH_{2}\right]}^{3}.$$

**Table 1:** Heats of formation and entropy at standard conditions (25 °C, 1 bar).

Molecule		*H* (kJ mol^−1^)		*S* (J/mol K)		Abundance
$H_{2}\left(g\right)$		0		130.68 [19]		$1.1\times 10^{4}\;n_{\mathrm{CO}}$ [20]
${\mathrm{CH}}_{4}\left(g\right)$		−74.6 [21]		186.3 [21]		$10^{-3}\;n_{\mathrm{CO}}$ [22]
$C_{2}H_{2}\left(g\right)$		227.400 [21]		200.927 [21]		$3\times 10^{-4}∼10^{-3}\;n_{\mathrm{CO}}$ [23]
$H_{2}O\left(g,\mathrm{gas}\right)$		−241.83 [24]		188.84 [24]		$10^{-9}∼10^{-7}\;n_{H_{2}}$(for ortho-$H_{2}O$) [25]
CO(g)		−110.53 [24]		197.66 [24]		$n_{\mathrm{CO}}$
${\mathrm{CO}}_{2}\left(g,\mathrm{solid}\right)$		−427.4		51.07 [26]		-
${\mathrm{CH}}_{3}\mathrm{COOH}$		−433		282.84 [27]		$3.4\times 10^{-10}\;n_{H_{2}}$ [28]

The typical temperature of a molecular cloud is about 10–20 K, but temperatures of “warm clouds” are about 20–60 K, and molecular clouds with H II regions can reach 100 K [29]. So, we set 10K<T<100K in this article. The partial pressure of $H_{2}$, ${\mathrm{CH}}_{4}$, $C_{2}H_{2}$, $H_{2}O$, CO, and ${\mathrm{CH}}_{3}\mathrm{COOH}$ we use here are 70%, $6\times 10^{-8}$, $3\times 10^{-8}$, $7\times 10^{-9}$, $6.36\times 10^{-5}$, and $2.38\times 10^{-10}$, respectively. Utilizing these equations and parameters, we can compute the Gibbs free energy released for the methane and acetic acid production processes.

### 2.2. The Results

**Figure 1:**
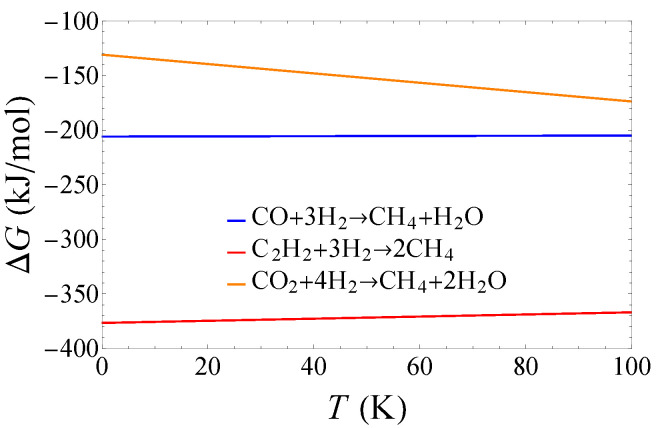
The Gibbs free energy released from the synthesis of methane.

**Figure 2:**
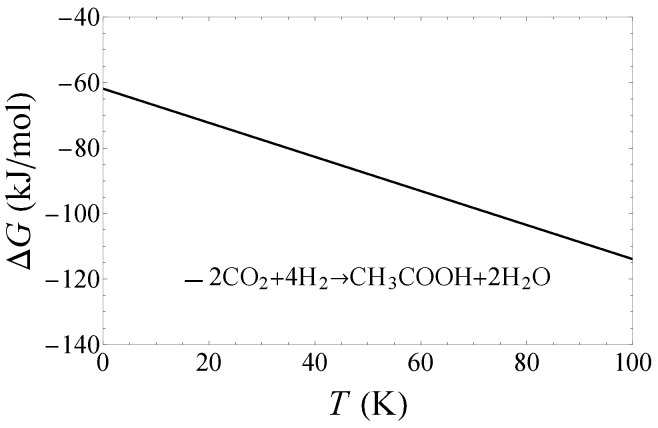
The Gibbs free energy released from the synthesis of acetic acid.

In Figure 1 and Figure 2, we show the Gibbs free energy released from the synthesis of methane (Figure 1) and acetic acid (Figure 2) in the molecular cloud environment. The change of Gibbs free energy is about −60–−370 kJ/mol. A negative value of Gibbs free energy is a spontaneous chemical reaction that releases energy. In Ref. [18], the authors measured the free energy values for four methanogens. They found that the minimum energy to sustain the growth of methanogen on Earth is about 8–15 kcar/mol (Table I in Ref. [18]). Following Ref. [12], we take an eigenvalue of 10 kcar/mol (i.e., 42kJ/mol) as the minimum Gibbs free energy required to maintain the survival of methane bacteria. From Figure 1 and Figure 2, we can see that the Gibbs free energies released by the reactions are energetically acceptable.

Normally, life forms require less energy at low temperatures and low-pressure environments. However, low pressure represents low number density, which profoundly influences the rate of chemical reactions. The density of acetylene is relatively low, and carbon monoxide is much denser. However, whether the reaction of acetylene and carbon monoxide can provide enough energy depends on the strength of metabolic activity at such low temperatures. Methanogenic and acetogenic life attached to solid carbon dioxide does not have the problem of insufficient raw materials. Solid carbon dioxide can provide sufficient energy and carbon sources for metabolic activities. In addition, there may be other bioenergetics mechanisms as a supplement, such as the mechanism driven by cosmic ray ionization [11]. Different bioenergetics mechanisms may work together. However, the processes discussed here have unique advantages in fixing carbon by converting carbon elements to organic molecules for life in molecular clouds using methane as an intermediate material.

In this model, the progeny of such methanogenic life would thrive in any solar system locale conducive to its existence, including Europa, Titan, and Mars, which possess dense carbon dioxide atmospheres. There has been an ongoing and intense debate about the detection of methane in Mars’s atmosphere. As detailed in Ref. [30], the authors have reported the discovery of methane in the Martian atmosphere, with a global average mixing ratio of approximately 10±5 parts per billion by volume, as measured by the Planetary Fourier Spectrometer aboard the Mars Express spacecraft. Comparable findings were presented in Refs. [31,32]. In Ref. [33], the researchers delved into both abiotic and biotic sources that can supply methane to Mars. However, other orbital observations have failed to detect methane in the Martian atmosphere, as noted in Ref. [34]. This issue still requires further research.

### 2.3. A Possible Distinguishing Signal

The consumption and production of carbon compounds through metabolic activities may significantly affect the distribution of these molecules. They can potentially serve as a trace signal of life within molecular clouds. According to this hypothesis, molecular cloud life uses solid carbon dioxide (carbon monoxide or $C_{2}H_{2}$) as a stable carbon source, which causes significant methane or acetic acid production. Consequently, it is reasonable to anticipate that the methane (or acetic acid) distribution within molecular clouds would mirror that of solid carbon dioxide. As the protostars form in a molecular cloud and the life forms therein potentially concentrate correspondingly, this distribution consistency would be more significant. If such a distribution consistency does not exist, it would be a great challenge for our model.

**Figure 3:**
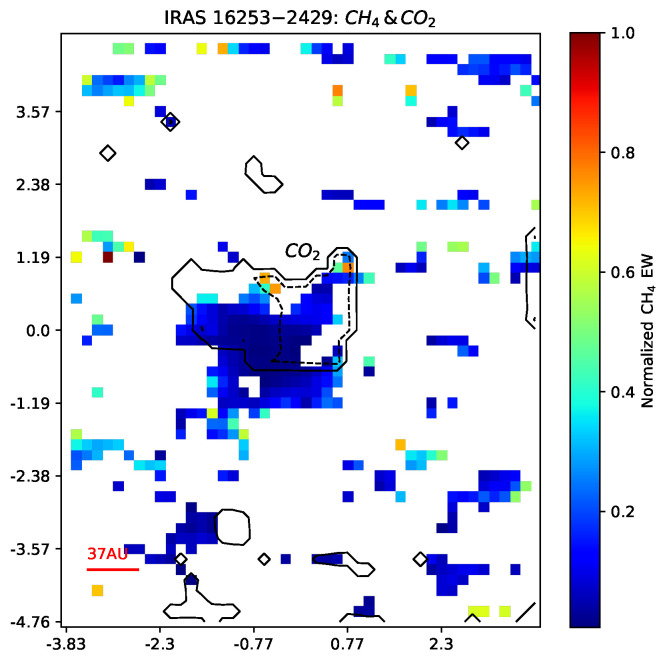
The distribution comparison between carbon dioxide and methane in IRAS16253−2429. The contour denotes the normalized equivalent width of ${\mathrm{CO}}_{2}$ with 0.1 (line) and 0.7 (dashed line). The data come from Ref. [37].

Some studies indicated that carbon dioxide and methane are distributed within the spatial region surrounding two protostars: IRAS 16253-2429 [35] and IRAS 23385+6053 [36]. After publishing the first version of this paper, we further analyzed the data from JWST. Our preliminary analysis indicates that the distribution consistency of carbon dioxide and methane does indeed exist in the region of protostars IRAS16253-2429 [37]. The comparative distribution of carbon dioxide and methane in the protostellar system IRAS16253-2429 is depicted in Figure 3 using the data presented in Ref. [37]. From this figure, it is easy to see that the distributions of carbon dioxide and methane closely align with each other.

This distribution consistency has a possible astrochemical origin, as it consists of the “Classical” dark-cloud chemistry model [38,39]. The “Classical” dark-cloud chemistry works in an environment of low temperature and gas density, where ion–molecule reactions dominate the carbon chemistry [38]. As shown in Figure 6 of Ref. [39], carbon dioxide and methane are distributed at the outer edge of the pre-stellar core according to the current observations, laboratory work, and modeling. Whether all or part of methane comes from methanogenic life is a primary research topic for our plans.

**Figure 4:**
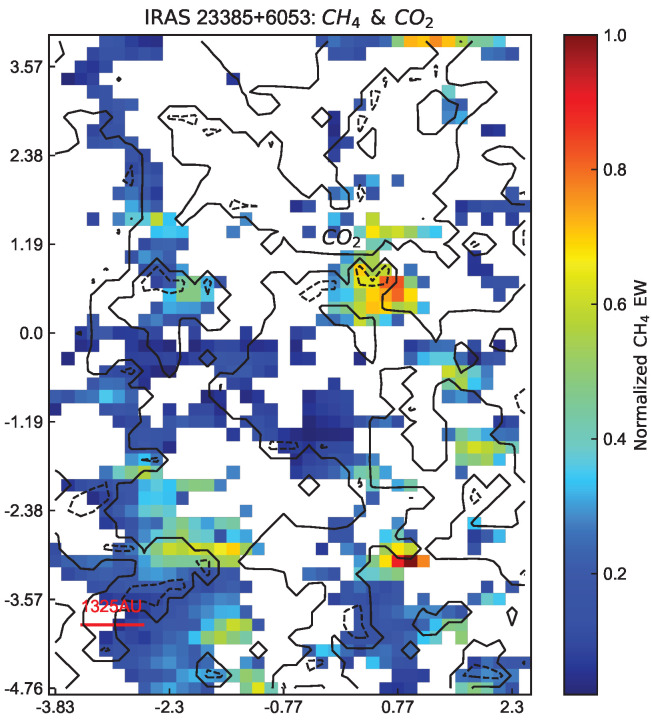
The distribution comparison between carbon dioxide and methane in IRAS23385+6053. The contour denotes the normalized equivalent width of ${\mathrm{CO}}_{2}$ with 0.1 (line) and 0.7 (dashed line). The data come from Ref. [37].

For protostars IRAS23385+6053, the distribution consistency is not very good as shown in Figure 4. It may be due to the instability induced by the accretion and turbulence processes of IRAS23385+6053 [40,41,42,43].

The same conclusion applies to the distribution of carbon monoxide and $C_{2}H_{2}$. We will further investigate whether the distribution of these molecules is consistent with methane in the future.

## 3. The Relationship with LUCA

If there were methanogenic or acetogenic life in the pre-solar nebula that then fell on the early Earth, LUCA would be one type of methanogenic or acetogenic life. Since acetogenic bacteria are usually considered a type of bacteria rather than archaea, LUCA may be more inclined toward methanogenic life. The three-domain tree of life presented by ribosomal RNA [44] depicted that LUCA is the last common ancestor of archaea, eukaryotes, and bacteria. Then, acetogenic life can still be a candidate for LUCA. Coincidentally, the primitive atmosphere of early Earth was rich in carbon monoxide and carbon dioxide [45,46]. These carbon oxides in the primitive atmosphere may have originated from volcanic eruptions but depend on the oxidation state of the upper mantle [47,48]. In addition, the impact of comets and asteroids may provide a large amount of CO [45,49]. This ensures the continued survival of methanogenic (acetogenic) life on Earth. Some previous studies [50,51] demonstrated that certain acetogenic and methanogenic Archaea utilized carbon monoxide as a carbon source during the early stages of Earth’s development.

The most important model prediction is that this molecular cloud life may be the predecessor of Earth’s life in space. More generally, LUCA is one type of life form that uses $H_{2}$ (CO or ${\mathrm{CO}}_{2}$) as an electron donor (acceptor) in the chemical reactions of generating energy. In general, this model provides a perfect origination of LUCA. There is already some tentative evidence about the probability that LUCA is one type of methanogenic or acetogenic life. Some research indicates that early lifeforms invented methanogenesis in the cooler temperature zones that led to the rise of LUCA after acclimatization to the hydrothermal vent environment by identifying the minimum genome of LUCA [52]. It was reported that LUCA’s genes point to methanogenic and acetogenic roots by surveying nearly two thousand genomes of modern microbes [53]. Despite a few challenging studies [54], research on geological evidence and phylogenomic reconstructions supports this thesis [55,56].

It should be noted that there is no suggestion here that life originated in molecular clouds. We believe that molecular cloud life may have derived from other planets in the Milky Way or planets in the solar’s predecessor star system [3].

## 4. Summary

This paper explores the possibility of methanogenic and acetogenic life in molecular clouds. The calculations demonstrate that the reaction of carbon monoxide, carbon dioxide, or acetylene with hydrogen molecules releases sufficient Gibbs free energy to ensure the survival of molecular cloud life. Solid ${\mathrm{CO}}_{2}$ may be the primary energy and carbon source because of its high local density. As the second most abundant molecule, the reaction of carbon monoxide is also a good option for providing energy. Additionally, we propose a potential distinguishing signal that can either support or disprove this model. We also consider the possibility that these methanogenic and acetogenic life forms could have been LUCA’s interstellar predecessor. Even now, their descendants and fossils may still be widespread in the solar system.

## Data Availability

No new data were created.
